# Fibrillary Glomerulonephritis Associated With Hashimoto’s Thyroiditis

**DOI:** 10.7759/cureus.72736

**Published:** 2024-10-30

**Authors:** Kevin T Dao, Arian Ashrafi, Matthew Palmbach, Tanya Eftekhari, Erfan Fallahtafti, Cynthia C Nast, Sabitha Eppanapally

**Affiliations:** 1 Internal Medicine, University of California, Los Angeles (UCLA)Kern Medical, Bakersfield, USA; 2 Internal Medicine, University of California, Los Angeles (UCLA) Kern Medical, Bakersfield, USA; 3 Pathology, Cedars-Sinai Medical Center, Los Angeles, USA; 4 Nephrology, University of California, Los Angeles (UCLA) Kern Medical, Bakersfield, USA

**Keywords:** associated, autoimmune disease, cyclophosphamide, dnajb9, fibrillary glomerulonephritis, hashimoto’s thyroiditis, immunosuppressive medications, nonamyloid fibrillary glomerular disease, p-anca, rituximab

## Abstract

Fibrillary glomerulonephritis is a rare glomerular disease with some correlations between this condition alongside viral infections, malignancy, and autoimmune pathologies. However, the question regarding the pathogenesis is whether patients who develop fibrillary glomerulonephritis do so irrespective of these other pathologies or whether such pathologies induce fibrillary glomerulonephritis through an unknown mechanism. As such, attempts have been made to create associations with this disease to demonstrate further understanding. Here, we would like to present a very rare case of a patient with Hashimoto’s thyroiditis associated with fibrillary glomerulonephritis and how such a diagnosis was made. Although autoimmune associations have been made alongside fibrillary glomerulonephritis, this case demonstrates one of the first cases of a patient with fibrillary glomerulonephritis and Hashimoto’s thyroiditis. A discussion regarding the treatment and management of our patient, as well as the pathogenesis of this disease, will also be included.

## Introduction

Fibrillary glomerulonephritis is generally classified as a type of nonamyloid fibrillary glomerular disease. The clinical presentation usually includes proteinuria, hematuria, renal function impairment, hypertension, and other nonspecific symptoms making a clinical diagnosis difficult [1]. To make a definitive diagnosis of this disease specifically, a kidney biopsy is needed. Various different types of microscopy, staining, and immunofluorescence have also been done in order to assess any discrepancies that this disease has in conjecture to other glomerular diseases [1,2-4]. Fortunately, many studies have noted a strong association with certain proteins as a marker to identify this disease. In fact, DNAJB9 was one such protein that was noted to be highly prevalent, specifically in fibrillary glomerulonephritis, but the reason as to why this protein was increased is still unclear [5]. What is also interesting is that this protein is highly specific and sensitive for fibrillary glomerulonephritis and isn’t seen in other glomerulopathies [5]. As of today, many other studies have attempted to assess associations between this disease and other pathologies to demonstrate a further understanding. One study that included 66 cases noted that 23% of patients had an underlying malignancy, with 17% having dysproteinemia and 15% with an autoimmune disease [6]. Of the patients with an autoimmune disease, the most common was Crohn’s disease, with an equal amount of patients having Graves' disease, idiopathic thrombocytopenic purpura, and systemic lupus erythematosus. Other autoimmune diseases such as Sjögren's syndrome, primary biliary cirrhosis, and ankylosing spondylitis were also noted [6]. However, recent literature reports little if any patients with fibrillary glomerulonephritis and Hashimoto’s thyroiditis. As such, we would like to present a rare case of a patient with both diseases as well as discuss the presentation, treatment, and management. A discussion regarding the pathogenesis and association of both diseases will also be included.

## Case presentation

A 58-year-old female with a past medical history of type 2 diabetes mellitus, hypothyroidism, pulmonary coccidiomycosis, and hypertension for approximately 10 years was referred to a nephrology clinic for concerns of chronic kidney disease G3b/A3 (Table 1). Initially at her primary care office, the patient had been noncompliant with medications for months due to running out of her medications for the past few months. She also noted some mildly orange-tinged urine but otherwise had no complaints or concerns at that current time. The physical exam was unremarkable, and repeat blood work was ordered. The patient’s medications were refilled, and the patient referred to nephrology for her chronic kidney disease and infectious disease for her pulmonary coccidiomycosis.

**Table 1:** Basic Metabolic Panel and Complete Blood Count BUN: blood urea nitrogen, GFR: glomerular filtration rate, TSH: thyroid-stimulating hormone, MCH: mean corpuscular hemoglobin, MCV: mean corpuscular volume

Parameter	Reference Values	Initial Presentation	1^st^ Appointment with Nephrology	2^nd^ Follow up appointment	3^rd^ Follow up appointment	4^th^ follow up appointment)	Other Subsequent Appointments
Sodium	137 – 145 (mmol/L)	136	136	139	136	138	137
Potassium	3.5 – 5.1 (mmol/L)	4.1	4.7	4.4	4.8	4.7	4.8
Chloride	98 – 107 (mmol/L)	105	105	111	106	112	110
Calcium	8.5 –10.1 (mg/dL)	8.9	9.1	9.4	9.7	8.7	9.2
BUN	7 – 18 (mg/dL)	30	25	22	55	45	49
Creatinine	0.51 – 0.95 (mg/dL)	1.47	1.46	1.30	2.15	1.90	2.49
GFR	> 60 (mL/min/1.73 m^2^)	37	37	42	24	27	20
Albumin	3.4 – 5.0 (g/dL)	3.7	3.3	-	3.9	-	-
T4 Free	0.9 – 1.8 (ng/dL)	-	-	1	-	-	0.4
TSH	0.550 – 4.780 (mclnt Unit/mL)	-	>150	87.453	-	-	23.2
Hemoglobin A1c	4.2 – 6.3%	6.8	6.1	-	-	-	5.1
WBC	4.5 – 11 (x 10^3^/mcL)	8.4	7.3	8.9	8.2	7.3	7.1
Eosinophil Absolute	<0.7 x 10^3^/mcL	0.3	0.2	0.3	0.2	0.2	0.3
Hgb	11.1 – 15.4 (g/dL)	9.9	10.5	10.9	10.8	9.6	9.7
MCV	75.7 – 97.6 (fL)	111.3	108.8	100	93.7	96.4	100.3
MCH	27.1 – 34 (pg)	39.2	38.8	34.5	32.7	34.1	35.1
Platelets	150 – 450 (x 10^3^/mcL)	272	213	298	248	223	189

At the first nephrology appointment, the patient noted that her urine had become more reddish in color, but she otherwise had no complaints or concerns. The patient's repeat blood work was noted to have a thyroid-stimulating hormone (TSH) >150, but creatinine and glomerular filtration rate (GFR) were stable compared to prior. Her hemoglobin A1C was 6.1, which was an improvement based on prior labs (Table 1). A bladder and renal ultrasound was ordered as well as a urinalysis, and serum protein electrophoresis (SPEP) and urine protein electrophoresis (UPEP) were ordered (Tables 2, 3). Blood work was then repeated, and another follow-up appointment (Tables 1-3).

**Table 2:** Urinalysis (UA)

Parameter	Reference Values	Initial Presentation	1^st^ Appointment with Nephrology	2^nd^ Follow up appointment	3^rd^ Follow up appointment	4^th^ follow up appointment)	Other Subsequent Appointments
UA Glucose	<30 (mg/dL)	30	<30	<30	<30	<30	100
UA Bili	Negative	Negative	Negative	Negative	Negative	Negative	Negative
UA Blood	Negative	Moderate	Moderate	Moderate	Large	Large	Large
UA Proteins	Negative (mg/dL)	300	300	300	600	200	>600
U Albumin/ Creatinine Ratio	1-30 mcg/mg	-	-	6,792.8	5,714.3	3,152.7	4,172.1
UA RBC	0 – 2/HPF	>50	>50	10-20	5-10	10-20	20 -50
UA WBC	0 – 2/HPF	10 – 20	10-20	5-10	2-5	2-5	>50
Squamous Epithelials	<20/HPF	5 – 10	5 – 10	5 – 10	2 – 5	2 – 5	0 – 2
Hyaline cast	0 – 2/HPF	0 – 2	0 – 2	0 – 2	0 – 2	0 – 2	0 – 2

**Table 3:** Serum Protein Electrophoresis (SPEP) and Urine Protein Electrophoresis (UPEP)

Parameter	Reference Values	1^st^ Appointment with Nephrology
SPEP Protein	6.1 – 8.1 (g/dL)	6.6
SPEP Albumin	3.8 – 4.8 (g/dL)	3.6
SPEP Alpha 1 Globulin	0.2 – 0.3 (g/dL)	0.3
SPEP Alpha 2 Globulin	0.5 – 0.9 (g/dL)	0.7
SPEP Beta 1 Globulin	0.4 – 0.6 (g/dL)	0.4
SPEP Beta 2 Globulin	0.2 – 0.5 (g/dL)	0.3
SPEP Gamma Globulin	0.8 – 1.7 (g/dL)	1.1
UPEP Creatinine	20-275 (mg/dL)	52
UPEP Total Protein	5-24 (mg/dL)	451
UPEP Protein/Creatinine Ratio	24 – 184 microg/g	8673 micro grams/g OR 8.673

At the second follow-up appointment, the patient's urinalysis showed moderate blood with 300 proteins and 10-20 red blood cell count (RBC) as well as five to 10 white blood cell count (WBC). Urinalysis showed a urine albumin/creatinine ratio of 6,792.8 mcg/mg (Table 2). Bladder ultrasound was unremarkable, and renal ultrasound showed normal in shape, size, and position of the kidneys with no renal calculi, cysts, or lesions. Patient’s repeat blood work also showed improvement in kidney function with creatinine of 1.3 as well as GFR of 42 (Table 1). Patient's TSH had also improved to 87.453 with free T4 of 1 (Table 1). Patient’s microscopic hematuria (Table 2) was attributed to advanced diabetic nephropathy, and the patient was recommended to continue her compliance with her medications along with lifestyle modifications. She was scheduled for repeat blood work in several months a few days prior to the next follow-up appointment.

Several months later, at the third follow-up appointment, the patient’s repeat lab work showed an acute kidney injury with creatinine of 2.15 and a declining GFR of 24 (Table 1). Urinalysis also showed an improving albumin/creatinine ratio of 5,714.3 mcg/mg. UA blood was also noted to be large, with an increase in UA protein of 600 and UA RBC five to 10 (Table 2). The patient’s acute kidney injury was associated to hypovolemia with an underlying glomerulonephritis, and the patient was advised to increase fluid intake. An infectious disease panel as well as an anti-neutrophilic antibody panel was ordered, and the patient was recommended to follow up (Tables 4, 5).

**Table 4:** Infectious Disease Panel Ig: immunoglobulin, Ab: antibody, GC RNA: Gonorrhea ribonucleic acid

Parameter	Reference Values	Initial Presentation	1^st^ Appointment with Nephrology	2^nd^ Follow up appointment	3^rd^ Follow up appointment	4^th^ follow up appointment)	Other Subsequent Appointments
Cocci IgM	Nonreactive	Nonreactive	-	-	Nonreactive	-	Nonreactive
Cocci IgG	Nonreactive	Very weakly reactive	-	-	Weakly reactive	-	Weakly reactive
Cocci Ab Titer	<1:2 Titer	<1:2 Titer	-	-	<1:2 Titer	-	<1:2 Titer
Quantiferon	Negative	-	-	-	-	-	Negative
Nil	Negative	-	-	-	-	-	Negative
Mitogen-Nil	Negative	-	-	-	-	-	Negative
GC RNA	Negative	-	Negative	-	-	-	
Chlamydia RNA	Negative	-	Negative	-	-	-	-
Hepatitis A Ab Total	Non-Reactive	-	Negative	-	-	-	-
Hepatitis B Core Antibody	Non-Reactive	-	Negative	-	-	-	-
Hepatitis B Surface Antibody	Non-Reactive	-	Negative	-	-	-	-
Hepatitis C Antibody	Non-Reactive	-	Negative	-	-	-	-

**Table 5:** Anti Neutrophilic Antibody Panel ANA: antinuclear antibodies, ANCA: antineutrophil cytoplasmic antibodies, P-ANCA: perinuclear ANCA, C-ANCA: cytoplasmic ANCA

Parameter	Reference Values	Initial Presentation	1^st^ Appointment with Nephrology	2^nd^ Follow up appointment	3^rd^ Follow up appointment	4^th^ follow up appointment)	Other Subsequent Appointments
ANA	Negative	-	-	-	Negative	Negative	Negative
ANCA	Negative	-	-	-	P-ANCA	P-ANCA	P-ANCA
C-ANCA	<1:20	-	-	-	N/A	N/A	N/A
P-ANCA	<1:20	-	-	-	1:640	1:320	1:640
Atypical P-ANCA	<1:20	-	-	-	N/A	N/A	N/A

**Figure 1:**
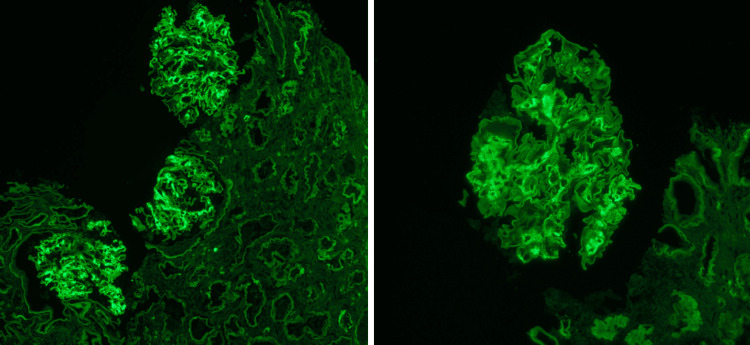
Immunofluorescence staining of kidney biopsy This figure shows immunofluorescence with mesangial staining for IgG

**Figure 2:**
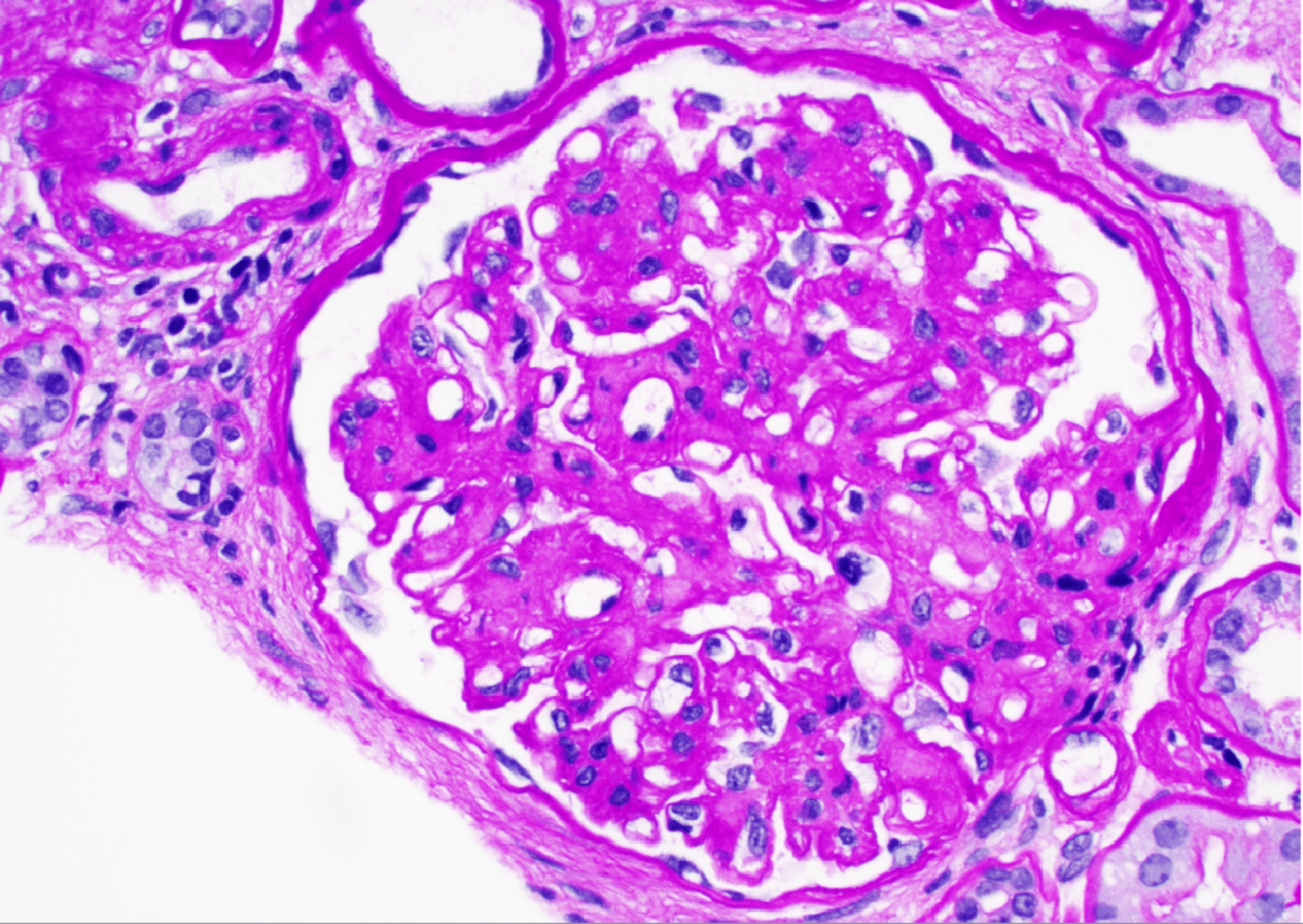
Periodic Acid–Schiff stain of kidney biopsy This Periodic Acid–Schiff stain of kidney biopsy displays mesangial matrix expansion with mild mesangial hypercellularity

**Figure 3:**
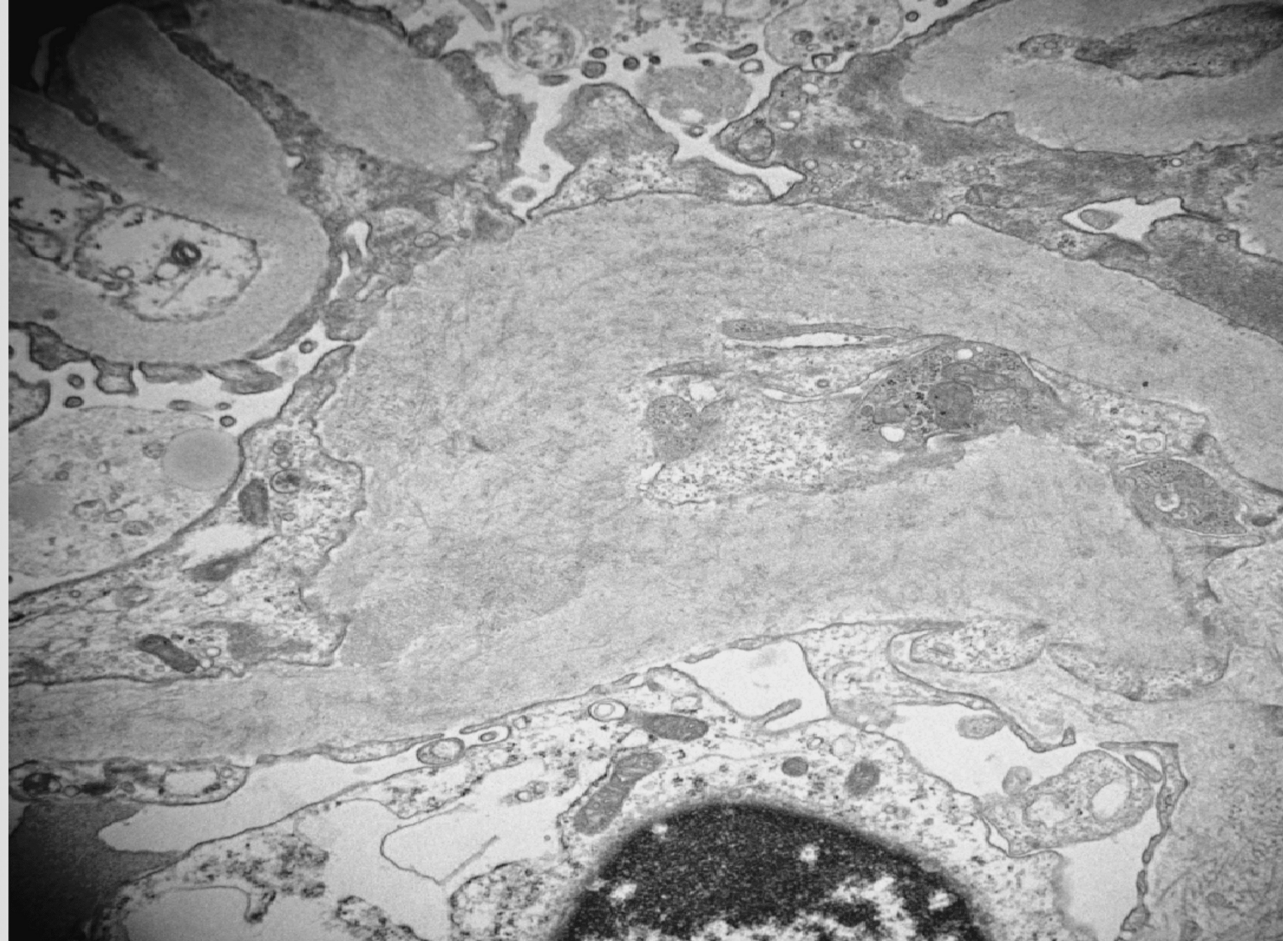
Electron microscopy of kidney biopsy The electron microscopy of the patient's kidney biopsy displays the mesangial areas expanded by abundant fibrils

**Figure 4:**
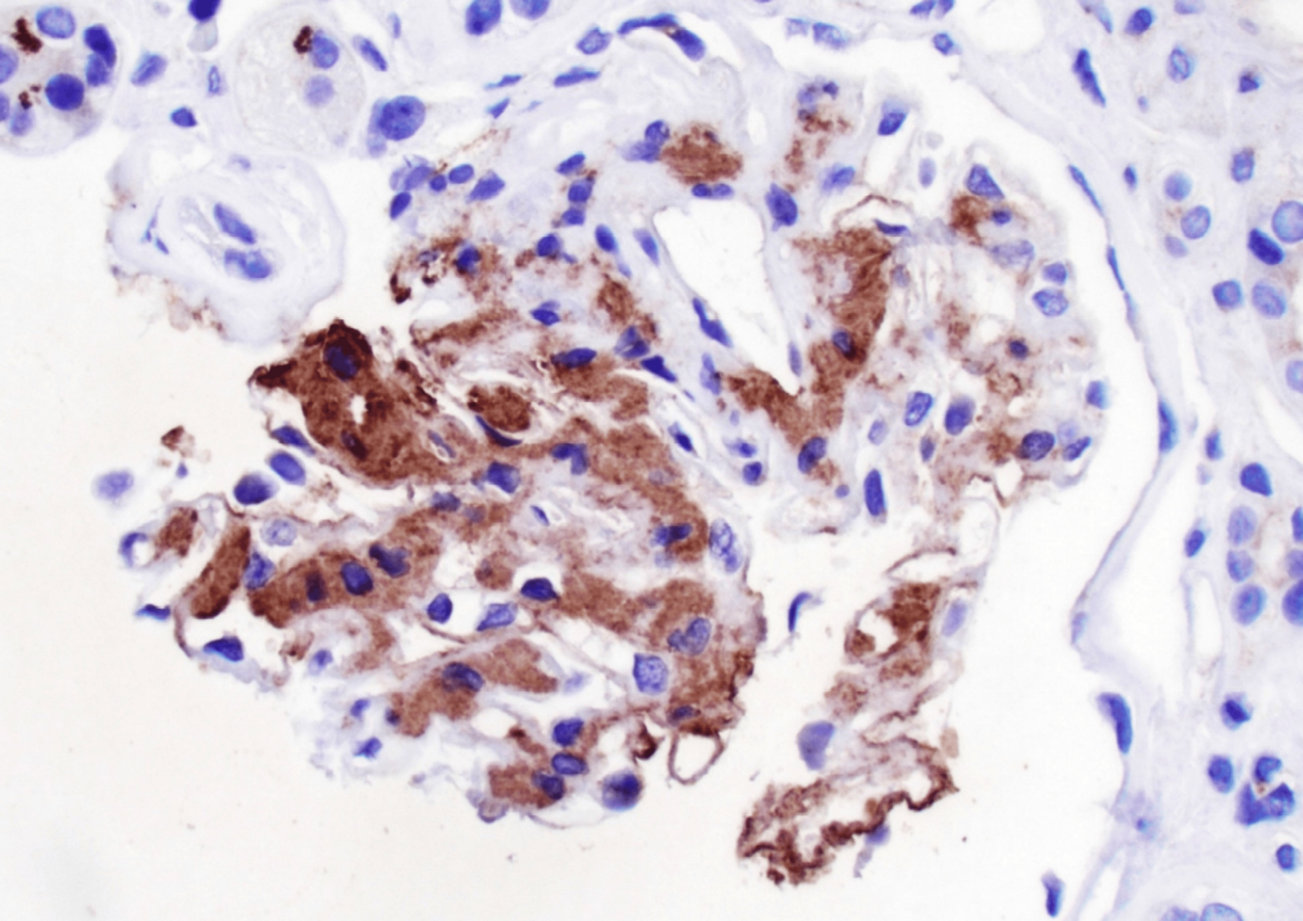
DNAJB9 staining of kidney biopsy The brown staining shows that there is positive DNAJB9

**Figure 5:**
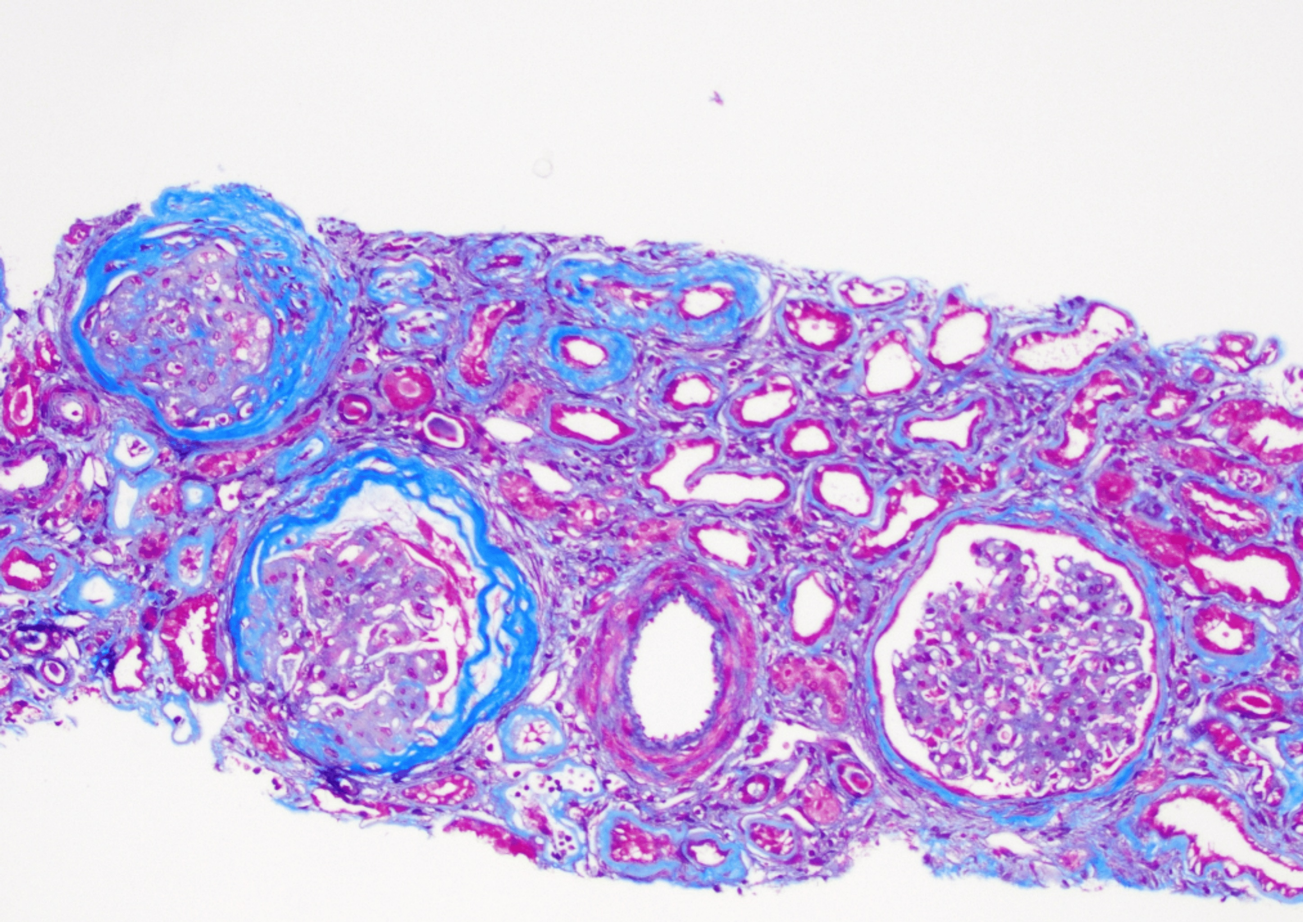
Trichrome Stain This stain shows severe tubulointerstitial scarring out of proportion to glomerular & vascular changes, consistent with superimposed chronic tubulointerstitial nephropathy

Blood work showed a positive perinuclear antineutrophil cytoplasmic antibodies (P-ANCA) with a titer of 1:640, but all other anti-neutrophilic antibody panels and hepatitis panel were unremarkable. However, she still noted to have an elevated, albeit improving, U Albumin/creatinine ratio, but creatinine and GFR were noted to be worsening, despite the patient not having any complaints and being compliant with her medications (Tables 1, 4-5). A kidney biopsy was then ordered, which showed fibrillary glomerular nephritis with positive DNAJB9 staining along with chronic tubulointerstitial nephropathy. Mild to moderate arteriosclerosis was also appreciated (Figures 1-5). A discussion was held with infectious disease and nephrology regarding starting immunosuppression. A full infectious disease panel was done, and the patient is currently pending her vaccinations with persistent observation of her kidney function.

At the fourth follow-up, the patient continued to endorse no significant complaints besides her orange-tinted urine, and an auto-immune panel was also done (Table 6). She was screened accordingly for tuberculosis before starting immunosuppression, and the autoimmune panel was noted to be unremarkable except for the patient having a significantly high thyroid peroxidase antibody of 102 units/mL. The patient’s kidney function showed mild improvement regarding creatinine, GFR, and U albumin/creatinine ratio, and tuberculosis panel was negative. At future upcoming appointments, the patient’s renal function began to decline with creatinine and GFR worsening. Infectious disease had then agreed that the patient was medically optimized to start cyclophosphamide for fibrillary glomerularnephritis and is starting treatment.

**Table 6:** Autoimmune Panel Ig: immunoglobulin, Ab: antibody, dsDNA: double-stranded DNA

Parameter	Reference Values	Initial Presentation	1^st^ Appointment with Nephrology	2^nd^ Follow up appointment	3^rd^ Follow up appointment	4^th^ follow up appointment)	Other Subsequent Appointments
C3	83 – 193 (mg/dL)	-	-	-	130	116	-
C4	15 – 57 (mg/dL)	-	-	-	37	35	-
dsDNA ab Crithidia	Negative	-	-	-	Negative	-	-
Glomerular Basement Membrane IgG	<1 (AI)	-	-	-	-	<1	-
SCL-70 Antibody	<1	-	-	-	-	-	<1
Sjogren’s Ab (SS-A)	<1	-	-	-	-	-	<1
Sjogren’s Ab (SS-B)	<1	-	-	-	-	-	<1
Thyroid Perxoidase-Ab	<9 Unit/mL	-	-	-	-	-	102

## Discussion

Fibrillary glomerulonephritis is generally a glomerular disorder with a variety of nonspecific symptoms; as such, the diagnosis is made with a kidney biopsy. Even so, this disease is still infrequently diagnosed since the diagnostic staining of this disease is only present in approximately 0.5 to 1.4% of kidney biopsies [3]. Light microscopy is not diagnostic since the findings would be similar to other glomerulopathies [3] However, electron microscopy would depict abundant fibillary deposits, as seen with our patient (Figure 3). Immunofluorescence would also display various protein staining such as C3, immunoglobulins, etc. (Figure 1) [2]. Nevertheless, the primary immunofluorescence with a positive staining for DNAJB9 would be highly specific for fibrillary glomerulonephritis (Figure 4) [5]. Once the diagnosis is made, the question regarding treatment should be discussed.

Due to the low prevalence of this disease, many trials have been done to find the optimal treatment for patients suffering from fibrillary glomerulonephritis. Previous studies that examined patients with fibrillary glomerulonephritis have noted that such patients tend to have other associated diseases as well, such as infection, malignancy, autoimmune, etc. [6]. However, whether or not these conditions are related to fibrillary glomerulonephritis through an unknown mechanism is still unknown. In patients with other disease associated with fibrillary glomerulonephritis, research has advocated for treating the patients other underlying disease or comorbidities in an attempt to slow down the progression of kidney disease. In doing so, it can serve to benefit the kidneys and prevent renal failure. In fact, one study noted that some patients with an underlying monoclonal gammopathy or lymphoproliferative disease with fibrillary glomerulonephritis show improvement in their kidney function while receiving treatment for their hematological/oncological disease. The same study noted that fibrillary glomerulonephritis seems to depict similar features to immunological disease [7]. This raises the question of whether immunosuppressive medications might play a strong role in treatment. Another study had enrolled 27 patients with fibrillary glomerulonephritis and noted that 13 of the patients’s had received immunosuppressive medication, with 46% having renal improvement. That study showed that the patients who had not received immunosuppressive medication developed chronic kidney disease, with a majority eventually developing end-stage renal disease [8]. Although only 46% of the patients who received immunosuppressive medication tend to show a response, there are some other studies that have shown the benefit of rituximab in treating dysproteinemias. In fact, there have been reports showing the benefits of using angiotensin-converting enzyme inhibitors, corticosteroids, and rituximab in treating immunoglobulin deposits in the kidneys. Unfortunately, said study only treated three patients with this regimen, but regardless, benefit was shown [9]. There has also been some research that tested corticosteroids and cyclophosphamide in two patients with fibrillary glomerulonephritis. The study noted a significant recovery in the renal function of both patients within six months, with one patient having a stable kidney function for approximately three and a half years [10]. Unfortunately, this is not always the case. In fact, there has been an instance where patients treated with cyclophosphamide and steroids did not prevent the progression of fibrillary glomerulonephritis to end-stage renal disease [11]. This in itself makes the conclusion of whether or not immunosuppressive medication would be beneficial since other studies had noted complete regression of this disease without any medications [6,12]. Overall, this shows the fickle nature of using immunosuppressive medications for fibrillary glomerulonephritis; unfortunately, however, they are the only treatments available.

Regarding our patient, she was noted to have hypothyroidism due to Hashimoto’s thyroiditis associated with fibrillary glomerulonephritis. Hashimoto’s thyroiditis itself is an autoimmune disease caused by lymphocytic infiltration as well as destruction of the thyroid gland. Due to this, patients tend to have symptoms of hypothyroidism however, there haven’t been many instances of this autoimmune disease affecting other organs directly but rather indirectly. This is because the thyroid itself is a metabolic organ with an effect on cholesterol, blood pressure, etc. which has been well documented and could have possibly resulted in indirect stress on her kidneys. While our patient also had other comorbidities that could have been associated alongside the glomerular disease, such comorbidities are less severe. On physical examination, the patient was noted to be unremarkable, while her diabetes was noted to be mild with an initial hemoglobin A1C of 6.8 with improvement on follow-up appointments (Table 1). The patient also had a history of pulmonary coccidiomycosis, but the fact that the patient was asymptomatic in regards to pulmonary issues with a very weakly reactive immune response and a <1:2 titer throughout all follow-up appointments made the association between pulmonary coccidiomycosis and fibrillary glomerulonephritis less likely.

The patient did have an elevated P-ANCA, which would be indicative of eosinophilic granulomatosis with polyangiitis and/or microscopic polyangiitis, but the suspicion of either was quite low. While the patient did have urinary indications such as proteinuria as well as microscopic hematuria, which could have supported either diagnosis, there were no other clinical symptoms that are classic of either disease. Regarding eosinophilic granulomatosis with polyangiitis, the patient denied any history of asthma, allergic rhinitis, chronic rhiոοѕinusitiѕ, peripheral neuropathy, weight loss, fevers, etc. [13]. The blood work also didn’t reveal any eosinophilia (Table 1), and the kidney biopsy didn’t show any necrotizing glοmеrսlοոephritiѕ or crescentic glοmеrսlοոeрhritiѕ, which is seen in both diseases [14-16]. Like eosinophilic granulomatosis with polyangiitis, the patient didn’t have any other symptoms that would indicate microscopic polyangiitis besides her urinary symptoms and urinalysis which are non-specific. Therefore, based on the history, blood work, and biopsy results, it is unlikely that either disease could have resulted in the patient developing progressive renal failure. It is more likely that the positive P-ANCA was more of an incidental finding. However, an argument can be made that there is an association between fibrillary glomerulonephritis and P-ANCA. Regardless, the fact that the patient started developing kidney failure due to fibrillary glomerulonephritis with the advent of severe uncontrolled hypothyroidism raises suspicion that there could be some correlation.

The fact that the patient had a positive thyroid peroxidase Ab approximately 11 times greater than normal with a TSH greater than 150 mclnt Unit/mL (Table 1) supported the association of Hashimoto’s thyroiditis and fibrillary glomerulonephritis. Of note the thyroid peroxidase Ab was done to show that the patient had Hashimoto’s thyroiditis rather than hypothyroidism secondary to an absence of thyroid gland or another unknown cause. Despite the association, the patient’s hypothyroidism improved due to treatment with levothyroxine (Table 1), but unfortunately her kidney function continued to decline. It raises the question of whether the patient’s underlying hypothyroidism could have triggered the development and/or progression of her fibrillary glomerulonephritis. While this is difficult to prove in and of itself, there does seem to be some correlation between the patient having severe hypothyroidism and the development of progressing kidney failure secondary to the fibrillary glomerulonephritis. Although this in itself can be coincidental, the fact that other autoimmune diseases have been associated with fibrillary glomerulonephritis raises the question of whether or not the patient’s Hashimoto’s thyroiditis could have played a role [6]. Regardless, the treatment was made to restart the patient’s levothyroxine, and a decision was made to start the patient on cyclophosphamide. The choice of immunosuppression was because cyclophosphamide had a more rapid response when compared to rituximab, and this was crucial due to the patient’s rapidly declining kidney function. While research is quite mixed on whether or not immunosuppressive medications show statistically significant benefit, it should still be heavily considered since approximately half of patients with this disease tend to rapidly progress to end-stage renal disease [17].

## Conclusions

Fibrillary glomerulonephritis is a fairly rare glomerular disease that has associations with autoimmune, hematological, infectious, and other pathological diseases. Although many autoimmune diseases have been shown to be associated with fibrillary glomerulonephritis, this is the first instance of severe hypothyroidism due to Hashimoto’s thyroiditis associated with fibrillary glomerulonephritis. Similar to other autoimmune diseases, the question still remains whether or not Hashimoto’s thyroiditis could have caused and/or progressed the patient's fibrillary glomerulonephritis, although proving a causation relationship would be difficult. Nevertheless, treatment should be geared to the associated disease to help stave off the progression of kidney disease; however, if kidney function continues to decline, immunosuppressive medications, particularly cyclophosphamide or rituximab, can be used. Even so, many studies have shown inconsistent benefits of immunosuppressive treatment; unfortunately, since not many other medications have been tested to treat fibrillary glomerulonephritis, these medications should be heavily considered. Overall, research regarding this disease is still heavily lacking, and hopefully further studies and treatments will help reveal more about this disease in time.
